# Visualization of cell-to-cell transmission of mutant huntingtin oligomers

**DOI:** 10.1371/currents.RRN1210

**Published:** 2011-02-11

**Authors:** Federico Herrera, Sandra Tenreiro, Leonor Miller-Fleming, Tiago Fleming Outeiro

**Affiliations:** Instituto de Medicina Molecular

## Abstract

We developed a new cell model for the visualization of toxic huntingtin oligomers in living cells. Huntingtin exon 1 (25Q or 103Q) was fused to non-fluorescent halves of the Venus protein. When huntingtin dimerizes inside the cells, Venus becomes functionally reconstituted and emits fluorescence. Oligomerization, aggregation and toxicity of mutant huntingtin were assessed by several procedures. We also present evidence that the transmission of huntingtin between cells can be determined in a quantitative manner with our model. Thus, this model can be a powerful screening tool for the identification of modifiers of oligomerization and cell-to-cell traffic of mutant huntingtin.

## **Introduction**

    Huntington´s disease (HD) is a progressive neurodegenerative disorder characterized by the loss of medium spiny neurons in the brain. This cell loss is associated with the misfolding and subsequent intracellular aggregation of a mutant form of huntingtin. The exon 1 of the IT-15 gene, which encodes huntingtin, contains the CAG repeats that are expanded over 35 in HD patients [1]. The resulting polyglutamine (polyQ) tract that is elongated over 35 glutamines in the mutant forms of huntingtin is especially important for the development of the disease, causing the misfolding, aggregation and toxicity of the protein [2] [3].

    Neuronal death seems to spread in a predictable manner in several neurodegenerative disorders [4] [5]. The traditional view is that neurodegeneration starts independently in different brain regions. The disorder would not actually spread from a brain area to another, but rather appear sequentially in different brain areas due to their different susceptibility to a particular insult. A more recent view considers misfolded proteins as prion-like “infectious” agents. According to this view, a misfolded protein could go from a sick cell to a healthy one, act as a seed for the generation of aggregates, and eventually kill the acceptor cell. Therefore, neurodegeneration could start in a primary focus and then spread progressively to other brain areas in a non-cell autonomous way, as it happens in prion disorders [6]. Although most evidence supporting this hypothesis comes from Alzheimer’s (AD) and Parkinson’s disease (PD) research fields, it has been shown that polyQ proteins such as huntingtin can be internalized by cells in culture [7] [8] [9]. Once inside cells, polyQ fibers act as a seed and are able to recruit soluble endogenous polyQ proteins.

    Huntingtin aggregates have variable size and degree of solubility. Growing evidence indicates that small huntingtin dimers and oligomers are more soluble and toxic than large aggregates, such as protofibrils, fibrils and inclusion bodies [10] [11] [12]. Due to their solubility properties, oligomers can diffuse more easily and act as seed for aggregation in neighbor cells. The understanding of how the aggregation process begins, i.e. how dimers and oligomers are formed, is therefore essential to identify possible molecular targets for the treatment of HD and other disorders involving protein misfolding and aggregation.

    In the present study, we developed a novel cell model for the visualization of huntingtin oligomers in living cells. In this model, based on the bimolecular fluorescence complementation assay (BiFC), huntingtin exon-1 is fused to non-fluorescent halves of the Venus fluorescent protein. When huntingtin fragments dimerize, the Venus halves get together and reconstitute the functional fluorophore. Fluorescence can then be measured by conventional methods, such as flow cytometry. Our model constitutes a novel system for the screening of genetic and pharmacological modifiers of huntingtin oligomerization, aggregation and toxicity. Furthermore, we present evidence that our model is a powerful tool for the study of cell-to-cell traffic of mutant huntingtin exon-1.

## **Results**

### Visualization of toxic mutant huntingtin oligomers by Bimolecular Fluorescence Complementation (BiFC) assay

         In order to find a combination of huntingtin-Venus BiFC constructs that reflects specific dimerization of mutant huntingtin, a series of plasmids carrying wild type (25Q) or mutant (103Q) huntingtin exon-1 fused to the non-fluorescent Venus halves were generated (Figure 1A). Huntingtin exon-1 was located at either N- or C-terminus of the Venus half 1 (V1, N-terminal half, amino acids 1-158) and at the N-terminus of the Venus half 2 (V2, C-terminal half, amino acids 159-238). A linker was included between huntingtin exon-1 and the Venus 1 half in one set of constructs in order to increase the flexibility of the fusion protein. A plasmid containing only the Venus 1 half but not huntingtin was also generated in order to have a control for spontaneous binding of Venus halves and for background fluorescence.          All possible combinations of plasmids were tested for fluorescence and generation of large intracellular aggregates 24 hours after transfection (Table I). The combination of plasmids showing higher average fluorescence as determined by flow cytometry was the one containing huntingtin exon 1 at the N-termini of both Venus halves (103QHtt-V1/103QHtt-V2). This pair was the only one that significantly leads to the formation of aggregates. All the other combinations showed a phenotype similar to the combination of Venus 1 with 25QHtt-V2 or 103QHtt-V2, suggesting that their binding is unspecific. Thus, Htt-V1/Htt-V2 combinations for both wild type and mutant huntingtin were chosen for the following experiments.

**Table I. **Outcome of the different combinations of Huntingtin-Venus BiFC plasmids in terms of fluorescence (oligomerization) and generation of larger aggregates.

**Table:** 

	Fluorescence			Aggregates		
	Second Half			Second Half		
First Half	Nothing	25QHtt-V2	103QHtt-V2	Nothing	25QHtt-V2	103QHtt-V2
Nothing	-	-	-	-	-	-
V1-Linker-25QHtt	-	+	+	-	-	-
V1-Linker-103QHtt	-	+	+	-	-	-
V1-25QHtt	-	+	+	-	-	-
V1-103QHtt	-	+	+	-	-	-
25QHtt-V1	-	+	++	-	+	++
103QHtt-V1	-	++	+++	-	++	+++
V1	-	+	+	-	-	-

Analysis of total cell lysates in denaturalizing conditions showed that cells actually carry the Htt-Venus fusion proteins (Figure 1B). However, when cells were transfected with the same amounts of DNA for both 25QHtt-Venus and 103Q-Venus constructs, wild type constructs were expressed at higher levels than the mutant ones, as previously described (Figure 1B, left blot) [13]. As a consequence, cells transfected with wild type constructs showed a mutant phenotype in terms of oligomerization, aggregation and toxicity (Data not shown). In order to balance expression levels cells were always transfected with wild type or mutant plasmid DNA in an approximate proportion of 1:6 (Figure 1B, right blot).

**Figure:**
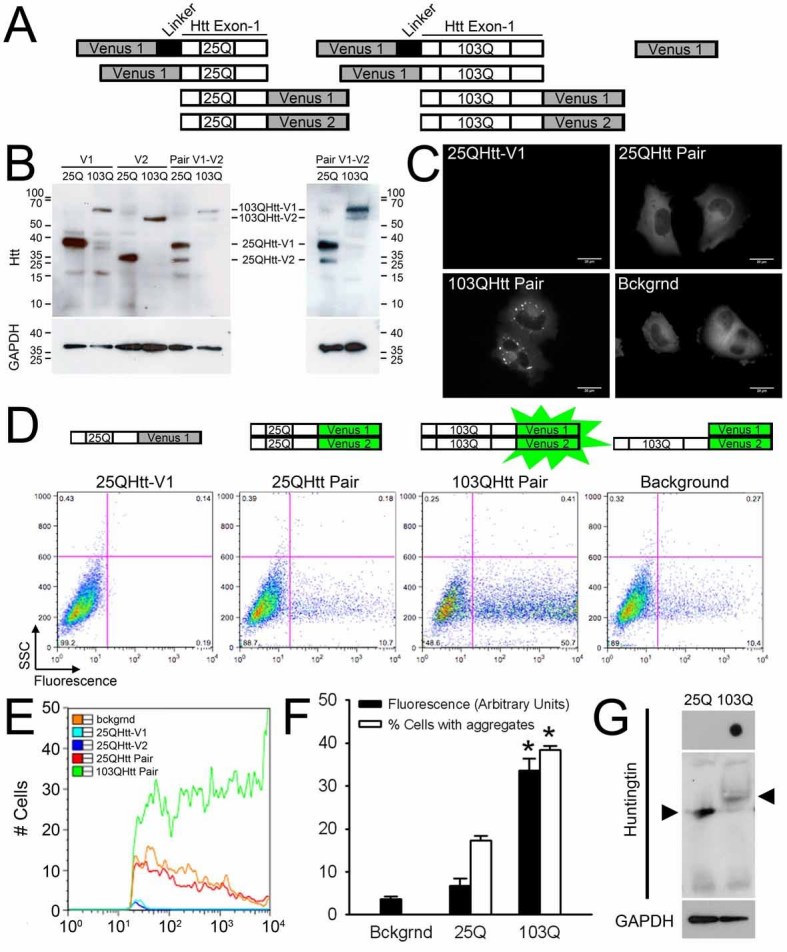
**Figure 1. **Visualization of mutant huntingtin (exon 1) oligomeric species by bimolecular complementation (BiFC) assay. A, Huntingtin-Venus BiFC constructs. B, SDS-PAGE immunoblot of total cell lysates shows the presence of huntingtin-Venus BiFC fusion proteins. Constructs containing 25Q huntingtin are expressed at approximately the same level than 103Q huntingtin constructs only when cells are transfected with DNA amounts at a 1:6 (25Q:103Q) proportion (right panel). C, Cells transfected with mutant huntingtin BiFC constructs show both diffuse fluorescence (dimers/oligomers) and larger aggregates. D, Transfection of H4 human glioma cells with the 103QHtt-Venus pair of plasmids causes an increase in the number of fluorescent cells and the average fluorescence versus control (25Q pair), as determined by flow cytometry. Transfection of cells with the 25QHtt-Venus pair of plasmids produces some fluorescence, at similar levels as background fluorescence observed when 103QHtt-V2 is combined with Venus 1 without huntingtin. Aggregates can be also found in cells transfected with wild type huntingtin, but at a much lower frequency. Cells transfected with Venus BiFC constructs without huntingtin show exclusively diffuse fluorescence. E, Representative histograms of flow cytometry experiments, superimposed for comparison. Mutant huntingtin shows more fluorescence than wild type huntingtin. F, Quantitative analyses of changes in average fluorescence and percentage of cells with aggregates upon transfection of cells with the BiFC constructs. *, significant versus 25QHtt pair, n=3, p<0.05. G, Filter trap assay (top) shows that 103Q huntingtin, but not the wild type form, generates SDS-insoluble aggregates. Native immunoblot of total cell lysates shows the generation of soluble oligomers by both mutant and wild type huntingtin.

Transfection of cells with 103QHtt-Venus constructs led to the generation of both diffuse fluorescence (oligomers) and large intracellular fluorescent aggregates (inclusion bodies) (Figure 1C). The localization of huntingtin oligomers and aggregates was mostly cytosolic, confirming previous reports (Rockabrand et al., 2007). No aggregates were observed in cells transfected with the control Venus 1 plasmid and the 103QHtt-V2 plasmid (Figure 1C, bottom right). As expected, 25QHtt- and 103QHtt-Venus halves were not fluorescent by themselves (Figure 1C-E). When cells were transfected with either 25QHtt- or 103QHtt-Venus pairs of plasmids, an increase in average fluorescence could be observed by flow cytometry, the 103QHtt-Venus pair being 5-fold higher than the 25QHtt-Venus pair (Figure 1D-1F). Cells transfected with the 25QHtt-Venus pair showed similar fluorescence levels to the cells transfected with a plasmid containing the control Venus 1 plasmid and a 103QHtt-V2 plasmid (background fluorescence) (Figure 1D-1F). However, background fluorescence photobleached very quickly upon exposure to light. On the other hand, 25QHtt-Venus fluorescence was significantly more stable (Figure 2). The presence of oligomers and large aggregates was confirmed by immunoblot analyses of protein extracts run under native conditions (Figure 1G, arrowheads) and filter trap assays (Figure 1G, top). Wild type huntingtin did not generate large insoluble aggregates, but readily formed dimers/oligomers. Interestingly, wild type huntingtin oligomers run as a single sharp band in native-PAGE gels, while mutant huntingtin oligomers formed a smear (Figure 1G, arrowheads).

**Figure:**
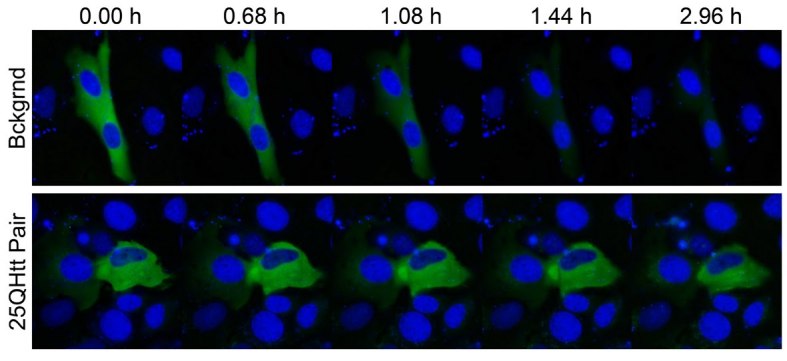
**Figure 2.** Background fluorescence photobleaches faster than wild type oligomers. Time lapse recording of cells transfected with control Venus 1 and 25QHtt-V2 plasmids (Bckgrnd) and cells transfected with the 25QHtt-V1 and 25QHtt-V2 pair of constructs (25QHtt pair). After only 30 minutes of recording, background fluorescence already starts to photobleach; and 1 hour later fluorescence disappears almost completely. On the other hand, wild type huntingtin oligomers show stable fluorescence for at least 3 hours.

Toxicity of mutant huntingtin constructs could be detected after only 24 hours (Figure 3A). In order to investigate the dynamics of oligomer formation and aggregation prior to cell death, cells transfected with 103QHtt-Venus constructs were analyzed by time-lapse microscopy. Recording started eight hours after transfection, because at this time point a few homogeneously fluorescent cells could be already observed by both flow cytometry and microscopy (Figure 3B). Figures 2C-2F shows a selection of representative cells that were recorded for 2-4 hours. Initially, cells had diffuse fluorescence and no aggregates were found (Figure 3C). Both cells showed small aggregates 30-45 minutes after starting the recording. After 1 hour, one of the cells showed a larger aggregate that grows quickly over time and scavenged the fluorescence from the rest of the cell, while the other did not generate larger aggregates. However, both cells eventually died. Dead cells always showed an apoptotic morphology, with condensation of the nuclei and membrane blebbing (Figures 3D-F), but they did not necessarily generate small or large inclusion bodies. Figure 3D shows a representative cell that died after showing small aggregates; Figure 3E shows two representative cells that died without showing aggregates; and Figure 3F shows a representative cell that produced a single large aggregate. It was also noticeable that, although large aggregates were translocated to the nucleus in some cases, this only occurred in late stages of the cell death process, when cells were already shrinking. Our interpretation is that large aggregates remain in the cytoplasm but superpose to the nuclei when cells shrink. The proportion of cells that die showing small or large aggregates was approximately the same as the proportion of cells that die without showing no aggregates (45.24 ± 5.87 % and 55.76 ± 7.58 %, respectively), suggesting that cell death is independent of the generation of aggregates and that oligomers are at least as toxic as larger aggregates. We also find very interesting that, when large aggregates were generated, the process was very fast and scavenged the fluorescence from the rest of the cell, indicating that the large aggregate is recruiting dimers and oligomers to its core.

**Figure:**
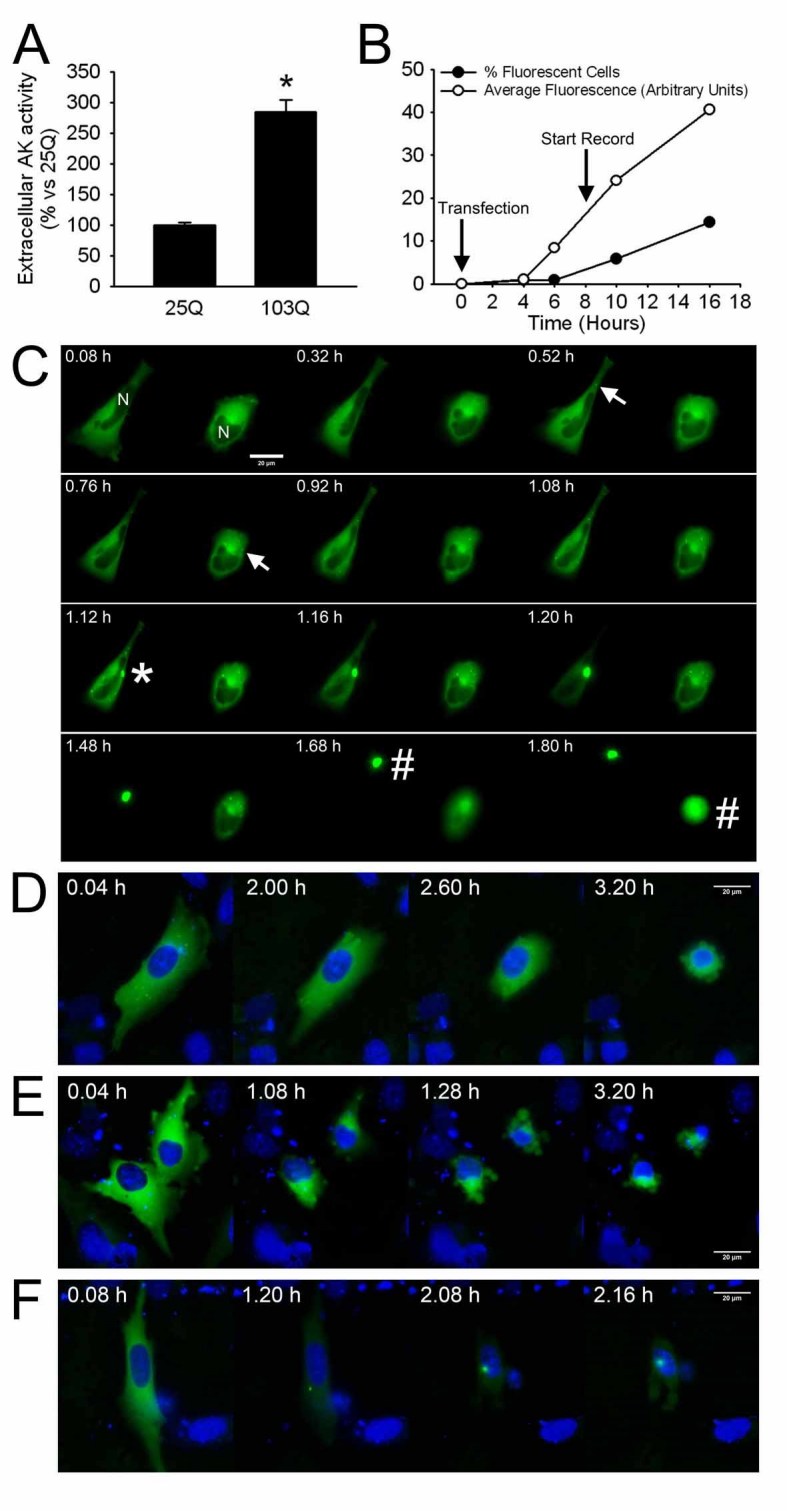
**Figure 3.** Mutant huntingtin oligomeric species are toxic. A, Mutant huntingtin induces a 2.7-fold increase in cell toxicity when compared with wild type huntingtin, as determined by the levels of extracellular AK activity. *, significant versus 25QHtt pair, n=3, p<0.05. B, Timeline of the appearance of fluorescent cells and the increase in average fluorescence as determined by flow cytometry. Fluorescent cells start to be seen on the microscope around 8 hours after transfection. Therefore, we started time lapse studies at this time point. C-F, Time lapse recording of representative H4 cells. In C: N, nucleus; white arrows, first small aggregates; *, a large aggregate starts to be generated; #, cells are death. In D-F: Nuclei are stained with DAPI (Blue). In all cases, scale bars are 20 µm.

### Determination of cell-to-cell traffic of mutant huntingtin by BiFC

    Expression of mutant huntingtin in astrocytes or other non-neuronal cell types can cause the death of surrounding neurons, an effect known as non cell-autonomous neurodegeneration [14] [15]. This effect could be caused by prion-like properties of some mutant and misfolded proteins, such as the ability to move from cell to cell and act as “seeds” that would prime the cells for further aggregation [6] [7]. We hypothesized that we could use our system for the study of the dynamics of cell-to-cell traffic of mutant huntingtin (Figure 4A). We transfected independent H4 or HEK cells with 103QHtt-V1 or 103QHtt-V2 plasmids and, 24 hours later, cell populations were mixed in equal proportions or seeded again independently.  Three days later, cells were analyzed by flow cytometry for the presence of fluorescent cells, which would indicate the existence of cell-to-cell traffic of mutant huntingtin (Figure 4B). Cells containing no plasmids were used as a reference to establish the baseline. The mixed population showed a significant number of fluorescent cells, indicating that there is traffic of mutant huntingtin between cells. On the other hand, cells containing a single BiFC plasmid showed a few or no fluorescent cells, a profile very similar to cells with no plasmid.

**Figure:**
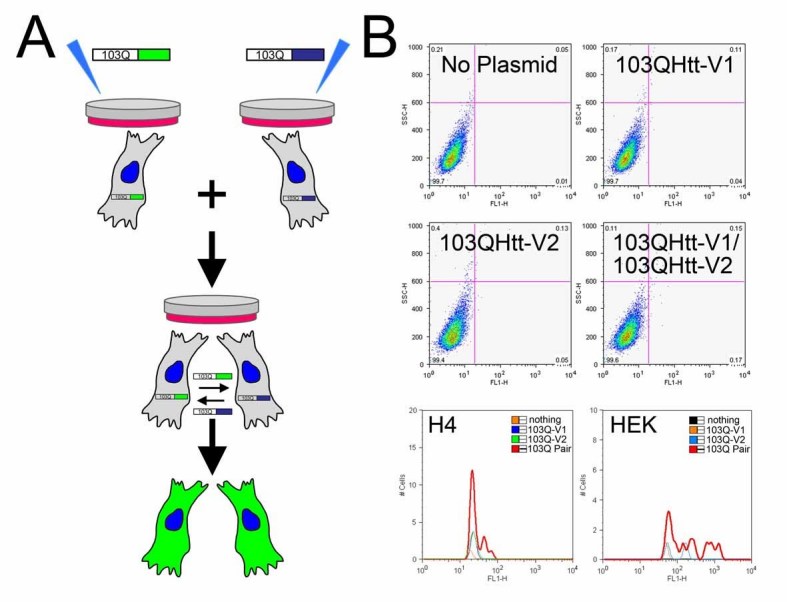
**Figure 4**. Quantitative analyses of cell-to-cell traffic of mutant huntingtin by BiFC. A, experimental design. Cells were transfected independently with either the 103QHtt-V1 plasmid or the 103QHtt-V2 plasmid. In the following day, these cell populations were mixed and left in co-culture for 3 days. If there is traffic of mutant huntingtin between cells, some fluorescent cells should be detected. B, Flow cytometry analyses of H4 and HEK cells show that there is some low rate movement of huntingtin between cells. Cells with no plasmid or with only one of the BiFC constructs were used as a reference.

## **Discussion**

    We developed a cellular model for the visualization and study of huntingtin oligomerization in living mammalian cells. This model is based on the BiFC assay. Huntingtin exon 1 was fused to non-fluorescent halves of the fluorescent protein Venus. When huntingtin dimerizes, the two halves get together and reconstitute the functional fluorophore. Similar models have been recently developed in the last few years for the study of the oligomerization of other aggregate-prone proteins [16] [17].

    Very recently, a huntingtin exon 1 BiFC-based model was described using split halves of the green fluorescent protein (GFP) [13]. Our results confirm and extend the reported observations. Both huntingtin BiFC models allow us to distinguish dimers and oligomers (diffuse fluorescence) from monomers (non-fluorescent) and inclusion bodies (large aggregates). In both cases, specific fluorescence was detected only when the fluorescent proteins were fused to the same end of the two interacting huntingtin fragments, suggesting that huntingtin assembles in parallel to form oligomers. However, Venus is one of the brightest fluorescent reporters from the third generation of fluorescent proteins, and it allows to carry out the BiFC assay without pre-incubating cells at 30ºC [18]. This is very important, because exposure of cells to lower temperatures triggers many cell responses that could add noise and greatly difficult the interpretation of results.

    We observed that wild type huntingtin exon 1 can generate oligomers by itself or oligomerize with mutant huntingtin (Table I) generating mixed phenotypes. However, we also showed that wild type oligomers are different and more homogeneous than mutant oligomers (native gel, Figure 1G).  Lower expression levels in cells transfected with mutant huntingtin constructs were observed than in cells transfected with wild type constructs [13]. In our system, this phenomenon caused wild type constructs to have a mutant phenotype, i.e. they showed the same levels of large aggregates, fluorescence and toxicity than mutant constructs. We demonstrated that, when expression levels are similar, wild type constructs barely generate large aggregates and the levels of fluorescence and toxicity are much lower than mutant constructs. An interesting question arises from our results: are there phenocopies of HD where huntingtin is not mutated but overexpressed? In this sense, duplications and triplications of the gene encoding alpha-synuclein underlie some forms of PD and Lewy Body disease [19] [20]; and individuals with a trisomy of chromosome 21, which encodes the amyloid precursor protein, develop AD [21] [22] [23].

    Our results indicate that mutant huntingtin oligomers are toxic and that inclusion bodies are neither necessary nor sufficient to induce cell death, supporting previous observations [10] [11]. While approximately one half of the cells that die show inclusion bodies, the other half do not show aggregates of any size before dying. Dead cells show nuclear condensation and blebbing of the cell membrane, a typical apoptotic morphology. Consistently, activation of caspase-3 has been reported in cells transfected with mutant huntingtin-GFP BiFC constructs [13].    Time-lapse microscopy showed that inclusion bodies are quickly generated, inducing at the same time a decrease in the fluorescence of the rest of the cell body. Our interpretation is that inclusion bodies recruit oligomers, and not only monomers, to their core. Large inclusion bodies barely move until late stages of cell death, when cells shrink. Both oligomers and inclusion bodies are usually located in the cytoplasm. We have not detected translocation of either oligomers or inclusion bodies into the nucleus during the cell death process. Although the first 17 amino acids of mutant huntingtin exon 1 keep the protein out of the nucleus [24], various post-translational modifications could trigger its transport to the nucleus by either active or passive paths [25] [26]. Most reports indicate that nuclear translocation of mutant huntingtin is necessary for cell death, but there seems to be a lack of correlation between the generation of intranuclear inclusions and cell death [2] [10] [25].

    Our model can be used not only to identify pharmacological and genetic modifiers of huntingtin oligomerization and toxicity, but also to study the traffic of mutant huntingtin exon 1 between cells. Growing evidence indicates that non-cell autonomous neuronal degeneration is an important process in all neurodegenerative disorders involving protein misfolding and aggregation, including HD [6] [27] [28]. First, supporting glial cells also carry mutant huntingtin and they do not die, but they can cause neuronal death by unknown means [14] [29] [30]. Second, misfolded proteins, including polyQ proteins, seem to have some weak prion-like properties. They can diffuse to the extracellular space and inside neighbor cells and act as seeds for further aggregation and neurotoxicity [6] [7] [8]. In this sense, monomers and oligomers diffuse much more easily than large inclusion bodies and, therefore, they are better candidates for the “infection” of neighbor cells. These properties could explain the regular spreading pattern observed in many neurodegenerative disorders. Our model could be a powerful tool for the study of the dynamics of mutant huntingtin between cells and to improve the understanding of the non-cell autonomous component of huntingtin neurotoxicity.

## **Materials and Methods**

### Cell cultures

    Culture dishes and flasks were purchased from Techno Plastic Cultures AG (Switzerland), with the exception of the poly-D-lysine-coated, glass bottom 35 mm dishes used for microscopy studies (Nr. 1.5, 10 mm glass surface diameter, MatTek Corporation, Ashland, MA, USA). Human H4 glioma cells were purchased from ATCC (HTB-148, LGC Standards, Barcelona, Spain) and maintained in OPTI-MEM I (Gibco, Invitrogen, Barcelona, Spain) supplemented with 10% FBS and a 1X antibiotic mixture (penicillin, streptomycin). For all the experiments, cells were seeded at a density of 10.500 cells/cm^2^. Cells were seeded on different types of plates depending on the application: 6-well plates (35 mm) for flow cytometry and toxicity assays, glass bottom 35 mm dishes for microscopy, and 60 mm dishes for protein extraction. Cells were transfected with the different combinations of plasmids using Fugene 6 tranfection reagent (Roche diagnostics, Mannheim, Germany) or lipofectamine 2000 (Invitrogen, Barcelona, Spain) 16-24 hours after seeding. In order to balance expression levels cells were always transfected with wild type or mutant plasmid DNA in an approximate proportion of 1:6 (0.3 µg of wild type plasmids versus 2 µg of mutant plasmids). Samples were collected or analyzed 24 hours after transfection unless otherwise indicated. 

### Constructs

    Lentiviral plasmids containing normal (25Q) and mutant (103Q) huntingtin exon-1 were a kind gift from Dr. Flaviano Giorgini (Department of Genetics, University of Leicester, Leicester, United Kingdom). Huntingtin open reading frames were extracted from these plasmids by PCR and cloned into pCS2 plasmids containing the Venus N-terminal (V1, amino acids 1-158) or C-terminal (V2, amino acids 159-240) sequences.

### Flow Cytometry

    Cells were collected by trypsinization (5 min at 37 ºC), washed once with PBS, and fixed in 1 % (w/v) paraformaldehyde in PBS for 10 min at room temperature. Samples were analyzed by means of a FACSCalibur flow cytometer (Beckton Dickinson, Franklin Lakes, NJ, USA). Ten thousand cells were analyzed per group. Graphics and data analysis were carried out by means of the FlowJo software (Tree Star Inc., Ahsland, OR, USA).

### Microscopy

    Twenty-four hours after transfection, pictures of fluorescent cells were taken with an Axiovert 200M widefield fluorescence microscope equipped with a CCD camera (Carl Zeiss MicroImaging GmbH, Germany). Live imaging was carried out in small stage incubator at 37ºC and 5% CO_2_. Images were analyzed and prepared for publication by means of the ImageJ free software (http://rsbweb.nih.gov/ij/).

### Immunoblotting

    Twenty-four hours after transfection, proteins were extracted by scrapping cells directly from the plates into lysis buffer. For denaturalizing conditions, the lysis buffer was 0.1% Triton X-100, 150 mM NaCl, 50 mM Tris pH 7.4 and a protease inhibitor cocktail tablet (Roche diagnostics, Mannheim, Germany). For native conditions, the lysis buffer was 50 mM Tris-HCl pH 7.4, 175 mM NaCl, 5 mM EDTA and a protease inhibitor cocktail tablet (Roche diagnostics, Mannheim, Germany). Cells were then sonicated and centrifuged at 10,000 x g for 10 min at 4 ºC. Supernatants were collected and the protein concentration was quantified by the Bradford assay (Bio-rad, Hercules, CA, USA). Fifty micrograms of protein were loaded and run on 12 % acrylamide SDS-PAGE for denaturing conditions, or on miniProtean TGX 4-20% acrylamide gradient precast gels (Bio-rad, Hercules, CA, USA) for native conditions. Proteins were transferred to PVDF membranes for blotting with antibodies against Huntingtin (1:1000, Millipore, Billerica, MA, USA), GAPDH (1:30000, Ambion, Austin, TX, USA) as indicate in each case. Immunoblots were developed with enhanced chemiluminescence reagents (Millipore, Billerica, MA, USA) and exposition to autorradiographic film.

### Filter retardation assays

    Cell lysates were obtained as described above in denaturalizing conditions and mixed with 1% SDS. A total of 100 mg of protein extract was loaded in a dot blotting apparatus and filtered by vacuum in acetate cellulose membranes (0.22 μm pore; GE Water & Process Technologies, Fairfield, CT, USA). Slots were washed two times with PBS 1%SDS. Detection of huntingtin in the filter was done by western blot as described above.

### Toxicity assays

    Cells were plated in 6-well plates and transfected 16 hours later with different combinations of plasmids. Toxicity was determined 24 hours after transfection by means of the Toxilight assay (Lonza Rockland Inc., Rockland, ME, USA), following manufacturer´s instructions. Luminescence was read in a microplate luminometer (Tecan).

### Statistics

    All graphs show the average ± standard deviation of at least 3 independent experiments. Statistical analyses were carried out by means of the SigmaPlot 11.0 software (Systat Software Inc., Chicago, IL, USA). Experiments comparing 2 groups were analyzed by a t-test; and experiments including more than 2 groups were analyzed by means of a one-way ANOVA followed by a Tukey post-hoc test. Statistical significance was only accepted when p<0.05.

## **Funding information**

TFO is supported by an EMBO Installation Grant and a Marie Curie International Reintegration Grant (Neurofold). FH, LMF and ST are supported by fellowships from the Fundação para a Ciência e a Tecnologia (SFRH/BPD/63530/2009, SFRH/BD/36065/2007, and SFRH/BPD/35767/2007, respectively).

## **Competing Interests**

The authors have declared that no competing interests exist.

## **Affiliations and contact Information**

Federico Herrera^1^, Sandra Tenreiro^1^, Leonor Miller-Fleming^1^ and Tiago Fleming Outeiro^1,2,3^* ^1 ^Cell and Molecular Neuroscience Unit, Instituto de Medicina Molecular, Lisboa, Portugal. ^2 ^Instituto de Fisiologia, Faculdade de Medicina da Universidade de Lisboa, Av. Professor Egas Moniz, 1649-028 Lisboa, Portugal. ^3 ^Department of Neurodegeneration, University Medicine Gottingen, Waldweg 33, 37073 Gottingen, Germany.   Correspondence should be addressed to: Dr. Tiago Fleming Outeiro, Phone: +(351) 21 799 9438; Fax: +(351) 21 799 9436; e-mail: touteiro@gmail.com
